# The competence of street food vendors to provide nutritious and safe food to consumers: a cross-sectional survey among street food vendors in Northern Ghana

**DOI:** 10.1017/jns.2023.65

**Published:** 2023-07-25

**Authors:** Mubaric Yakubu, Patience Kanyiri Gaa, Gabriel Libienuo Sowley Kalog, Victor Mogre

**Affiliations:** 1 Department of Nutritional Sciences, School of Allied Health Sciences, University for Development Studies, Tamale, Ghana; 2 Department of Health Professions Education and Innovative Learning, School of Medicine, University for Development Studies, Tamale, Ghana

**Keywords:** Competence, Food safety, Food safety attitude, Northern Ghana, Nutrition knowledge, Practice

## Abstract

Increasingly most people have their meals outside their homes and are vulnerable to illnesses caused by unsafe foods. Unsafe food preparation and supply by vendors have made food safety a concern for public health. The present study evaluated the nutrition knowledge, attitude and food safety and hygienic practices of street food vendors (SFVs) in Northern Ghana. An analytical cross-sectional study design was conducted among 424 SFVs, and the data were collected using questionnaires and observation. The mean ± sd nutrition knowledge score of the SFVs was 7⋅08 ± 1⋅75 in which the majority of the participants (68⋅6 %) knew foods that help fight diseases and build immunity. The mean ± sd food safety and hygienic practice score was 7⋅61 ± 2⋅66 with more than half of the participants reportedly not using hand gloves while preparing and serving food. Factors that were associated with food safety and hygienic practices of the SFVs were level of education (*β* = −0⋅36, *P* < 0⋅001), number of hours worked (*β* = 0⋅15, *P* = 0⋅002), food hygiene and safety knowledge (*β* = 0⋅21, *P* = 0⋅002), having a business certificate (*β* = −0⋅15, *P* = 0⋅004) and having medical check-up (*β* = 0⋅11, *P* = 0⋅029). The food safety and hygienic practices of the SFVs may constitute a food safety risk to consumers. Improving food safety and hygiene knowledge may be important but regular monitoring and check-up by the FDA could result in SFVs following the required food safety and hygienic practices.

## Background

Foods that are edible and usually sold in the streets and places with less than four walls are considered to be street foods^(1)^. According to the Food and Agriculture Organization^(2)^, street food is defined as ‘already cooked foods and beverages sold on streets or other public places by hawkers’. Globally, an increasing number of people depend on street foods because of how it is easily accessible, affordable and convenient to many millions of people^(3)^. Every day, about 2⋅5 billion people consume foods on the street, which supports the livings of masses of low-income individuals, meeting their nutritional needs and at the same time contributing meaningfully to the economy^(4)^. Street foods provide benefits such as the provision of variability of low-cost, suitable food, diversified diets and delivery of employment and income particularly for women^(5)^. For many people in poor countries, food vendors provide a convenient diet. Streets foods have been recognised as a promising vehicle for micronutrient fortification due to being inexpensive, available and forming an integral part of the diet in many countries. In addition, street foods are also characterised by regularity, consistency and frequency of consumption in all income groups especially the urban poor and children^(4)^.

Street food vendors (SFVs) are an imperative part of the ‘farm to plate’ food safety field, which is important for the prevention and management of foodborne illnesses^(3)^. The World Health Organization reported that nearly one person out of 10 people gets sick after eating contaminated food, and 420 000 people die each year from diarrhoeal-related diseases, subsequently leading to a loss of 33 million healthy life years^(6)^. Street foods have been frequently reported to contribute to foodborne illnesses and as a result, the safety of street foods has been given some major attention relating to microbial contamination, pesticide residues, transmission of parasites, the use of unpermitted chemical additives and environmental contamination^(7)^.

Given that street foods are important sources of diet for several people, it is necessary to determine the nutrition knowledge of SFVs as well as their food safety practices. However, most studies in the literature focus on the food safety knowledge and practices of SFVs with little research on the nutrition-related knowledge of these SFVs^(8)^. There is thus the need for research to focus on nutrition knowledge together with their food safety practices in order to inform the design of interventions aimed at improving the competencies of SFV to provide safe and nutritious foods to the public. We evaluated the nutrition knowledge, attitudes towards nutrition, as well as food safety and food hygienic practices of SFVs in the Tamale Metropolis in the Northern Region of Ghana. We also determined the factors associated with the food safety and hygienic practices of SFVs in the Tamale Metropolis.

## Methods

### Study area

The study was carried out among SFVs within the Tamale Metropolis of the Northern Region of Ghana. Being the capital of the region, its location offers it a viable market for economic activities drawing people from within and outside the region, including traders from some of the neighbouring sub-Saharan African countries enabling the proliferation of SFVs. It has a population of 374 744 inhabitants.

### Participants

An analytical cross-sectional research design was employed. The research was limited to SFVs in the Tamale Metropolis (i.e. the central business district of the metropolis, the University for Development Studies, the Tamale campus and its environs, Lamashegu, and the Tamale Teaching Hospital and its environs). Both stationary and mobile SFVs were eligible for inclusion. Food vendors on the streets and hawkers on bus terminals, private and government institutions’ premises (schools, hospitals, etc.) were eligible to participate in the study. However, hotels, restaurants and food manufacturing industries were exempted from the study.

### Sample size determination

The sample size is calculated using the Hawkins^(9)^ formula for a point estimate sample:
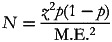
where *N* is the sample size; *z*^2^ is the abscissa of the normal curve that cut off an area at the tail (1 refers to the desired confidence level, 95 %) which is the critical value of 1⋅96; *p* is the estimated proportion of nutrition knowledge, attitude and practice by food vendors from similar study with a standard prevalence of 50 %; M.E. is the desired level of precision (5 % = 0⋅05):
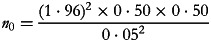
*n*_0_ = 0⋅9604/0⋅0025; *n*_0_ = 384⋅16.

Thus, the calculated sample size is approximately 385. Using a 10 % as the non-response rate, we generated a sample size of 424.

### Data collection procedures

Permission was sought from the Tamale Metropolitan Health Directorate and the Metropolitan Assembly to begin data collection. Due to the high number of SFVs in the metropolis, it was stratified into three zones: zone 1 (the central business district of the metropolis), zone 2 (University for Development Studies, Tamale campus and its environs and Lamashegu) and zone 3 (Tamale Teaching Hospital and its environs). SFVs in each zone who met the inclusion criteria were contacted at their various vending points. Data were collected with an online questionnaire designed using the KoboCollect software application. Prior to data collection, pre-testing was performed on ten SFVs, and feedback was used to improve the overall quality of the questionnaire. Three University graduates were trained as research assistants (RAs) for the purposes of data collection. The RAs were frequently supervised and monitored by the lead author. Due to the high numbers of SFVs, purposive sampling was employed by the RAs to include specific SFVs such as mobile vendors, and those who operate in open spaces and introduced the study to them and invited them to participate. Those who consented to participate were taken through the informed consent procedures and recruited into the study. The questionnaire was then administered to them in a secluded place near the vending point. Questions were read to the hearing of those who understood the English language, and those who could not speak and/or write in the English Language had the items translated into a local dialect, i.e. *Dagbanli* for them. No participant was forced to participate in the study. The methodology of this research conformed to the ethical principles of the Helsinki Declaration. Verbal and written informed consent was obtained from participants. Participants were assured that they were at liberty to withdraw from the study at any point if they felt uncomfortable. Ethical approval was obtained from the Committee on Human Research and Publication and Ethics (EHPE) of the Kwame Nkrumah University of Science and Technology (KNUST), (CHRPE/AP/191/21).

### Data collection methods

Data were collected using a questionnaire. The questionnaire evaluated the socio-demographic characteristics of the participants (such as age, sex, level of education, religion, marital status, training on food safety, business certificate and medical check-up). There were eleven items that assessed participants’ knowledge of nutrition using multiple-choice questions and to choose the most appropriate answer format. A correct answer was scored one mark and a zero mark was given for a wrong answer. The total nutrition knowledge scores were obtained by summing up the marks gained for each item for which the mean score was computed. A total of fourteen items assessed participants’ food safety knowledge. One mark was given for a correct answer and a zero mark was given for a wrong response. The total food safety knowledge scores were obtained by summing up the marks gained for each item for which the mean score was computed. Twelve items assessed participants’ attitudes towards food safety and hygienic practice. Each item was assessed using a 5-point Likert scale, i.e. 1: strongly disagree, 2: disagree, 3: neutral, 4: agree and 5: strongly agree. Strongly agree and agree were combined to constitute agree, and strongly disagree and disagree combined to constitute disagree. A 22-item checklist assessed participants’ food safety and hygienic practices. Participants were observed to see if they practiced food safety and hygiene. Those who followed any of the items identified in the checklist scored one and those who did not scored zero. The mean ± sd scores of food safety and hygienic practice of the SFVs were then computed by summing up the marks gained for each item. The items of the questionnaire were created by reviewing relevant literature and those of previously validated questionnaires (Addo-Tham *et al.*^(10)^; Akabanda *et al.*^(11)^; Hill *et al.*^(8)^).

### Data analysis

**Fig. 1.**
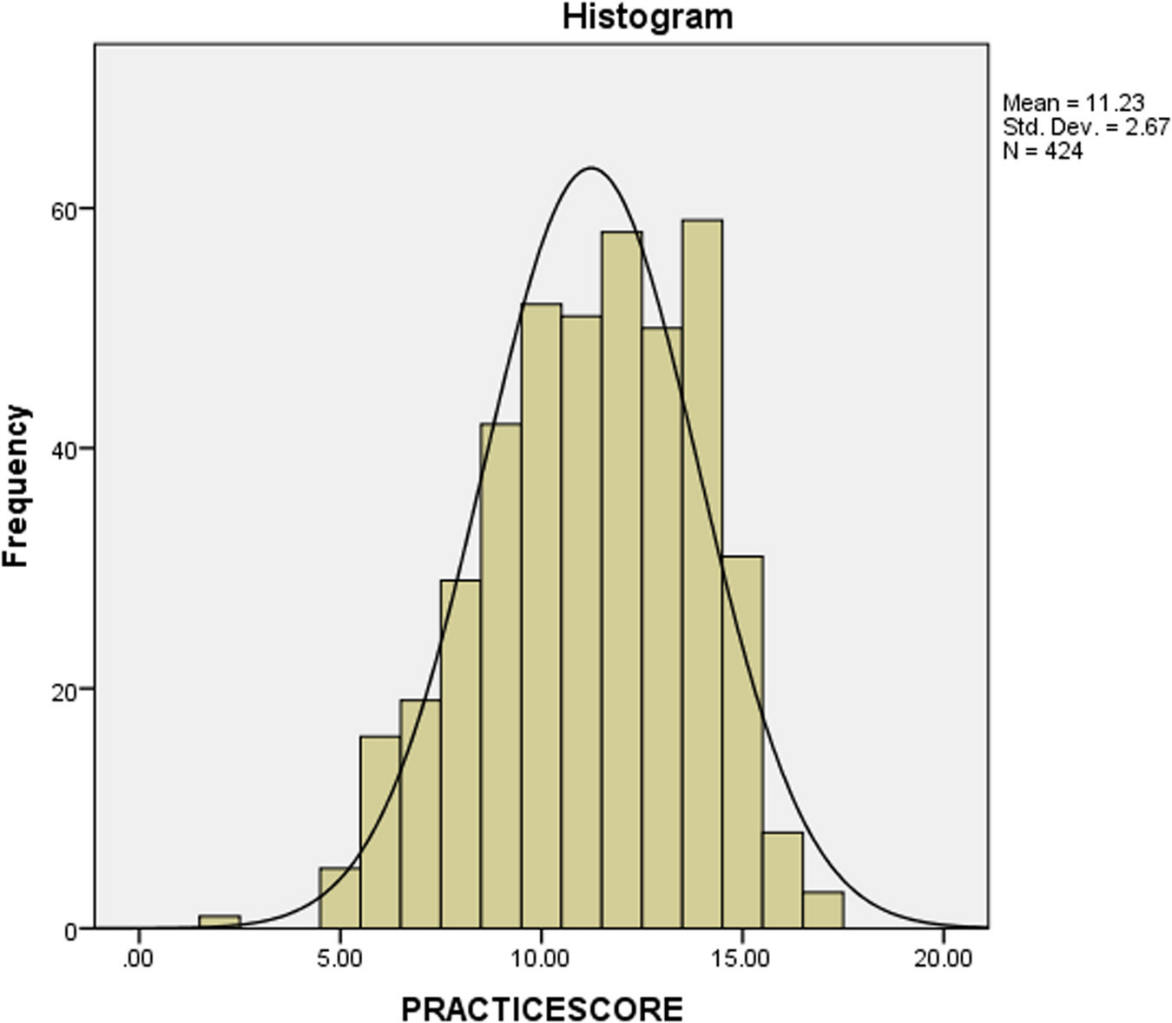
Normal distribution of food and hygienic practice scores of the participants.

Data were analysed using SPSS version 20. Descriptive statistics such as frequencies, percentages, mean scores and standard deviations were used to describe the data. Data were normally distributed upon examination of the histogram for the dependent variable (Fig. 1). Independent Student's *t* test and one-way ANOVA were used to test for association between categorical and continuous variables. A correlation was used to test for association among continuous variables. A linear regression model was used to determine factors associated with food safety and hygienic practice in which we adjusted for age, level of education, business certificate and other demographic characteristics. A *P*-value of less than 0⋅05 at a 95 % confidence interval was considered significant.

## Results

### General and socio-demographic characteristics of the participants

As shown in Table 1, 83⋅5 % of the participants were females and had a mean age of 33⋅10 ± 9⋅06 years (minimum = 10 years and maximum = 60 years). Also, 51⋅1 % had basic education, 57⋅1 % had not received training on nutrition and 52⋅8 % had no training on food safety.

**Table 1.** Socio-demographic and general characteristics of the respondents

Variable	Frequency (*n* 424)	Percent (%)
Gender		
Female	354	83⋅5
Male	70	16⋅5
Age (years)		
<20–30	169	39⋅8
31–40	164	38⋅7
41–50	81	19⋅1
50 and above	10	2⋅4
Level of education		
Low	217	51⋅2
High	86	20⋅3
None	121	28⋅5
Have you received training on nutrition		
No	242	57⋅1
Yes	182	42⋅9
Have you received training on food safety		
No	224	52⋅8
Yes	200	47⋅2

### Operational characteristics

SFVs sold starchy staples including banku (9 %), fried rice (8 %), kenkey (14 %) and waakye (18 %). The majority (87⋅7 %) of the SFVs did not have a business certificate, and 77⋅6 % did not undertake any form of medical check-up (Table 2).

**Table 2.** Operational characteristics of street food vendors

Variable	Frequency	Percent (%)
Has a business certificate to operate	52	12⋅3
Has undergone recommended medical check-up	95	22⋅4
Owns a business		
Joint owner	73	17⋅2
Non owner	77	18⋅2
Owner	274	64⋅6
Where is the food cooked		
Home	216	50⋅9
On-site	208	49⋅1
What happens to leftover food		
Thrown away	0	0
Taken home to eat	201	47⋅4
Given away	153	36⋅1
Sold the next day	70	16⋅5

### Nutrition knowledge of the participants

As shown in Table 3, greater than half of the participants (68⋅6 %) knew foods that help fight disease and build immunity, knew that excessive intake of salt could cause diseases (80⋅0 %) and that consumption of fruits and vegetables daily is good for a healthy heart (81⋅1 %). The mean ± sd nutrition knowledge was 7⋅08 ± 1⋅75 with a minimum score of 1⋅0 and a maximum score of 11.

**Table 3.** Nutrition knowledge of the respondents

Nutrition knowledge aspect tested	Frequency	Percentage
Foods that help fight disease and builds immunity	291	68⋅6
Knows energy giving foods	320	75⋅5
Foods that help to build the body	94	22⋅2
Foods that provides the body with blood	400	94⋅3
food that is good for a healthy heart	344	81⋅1
Foods that do not have added sugar	307	72⋅4
Knows a balanced diet	206	48⋅6
Knows diseases that could be caused due to high salt intake	339	80
Knows the negative effects of prolonged cooking	317	74⋅8
The following are some of the ways to preserve nutrient value of food except	59	13⋅9
Knows how nutrients can be lost	323	76⋅2

### Food safety knowledge

Sixty-one percent of the SFVs had knowledge about the importance of cooking food, 69⋅6 % knew that cooking food aids food to digest easily, 57⋅1 % believed cooking adds variety to food, 59 % said cooking adds flavour to the food and 55⋅9 % disagreed that cooking adds nutrients to food. Also, 87⋅3 % of the participants said poor personal hygiene could lead to food contamination, 64⋅9 % said time–temperature abuse can lead to food contamination, and 73⋅3 % said poor sales can lead to food contamination. The mean ± sd food safety knowledge score of the SFVs was 9⋅08 ± 3⋅14 with a minimum score of 2 points and a maximum score of 14 points.

### Attitudes of SFVs towards food safety and hygienic practices

In all, 80⋅4 % of the participants agreed good personal hygiene prevents foodborne infection, 72⋅4 % agreed that unsafe food could cause ill health, 80⋅7 % agreed that raw and cooked food should be stored separately to reduce the risk of contamination and 83 % agreed that safe food handling is an important part of their job responsibility. Only 46 % agreed that the reuse of oil may be harmful to one's health, 88⋅5 % said over-cooking can lead to loss of nutrients and 59⋅4 % agreed that diet diversity is an indicator of nutrient quality. The mean ± sd attitude of SFVs towards food safety and hygienic practice was 49⋅00 ± 9⋅60 with a minimum score of 12 points and a maximum score of 60 points.

### Food safety and hygienic practices of SFVs

The majority of the participants handled money and food simultaneously (78⋅8 %), 46⋅7 % had jewellery on hand while serving food and 48⋅8 % showed flu-like symptoms. Most of the participants had their hair neatly covered (61⋅3 %) but only about half of the participants used half aprons while preparing food (51⋅7 %), with only 37⋅7 % of the participants using full aprons. Greater than half of the participants did not use hand gloves while preparing and serving food, 88⋅9 % did not keep clean and short nails and 90⋅3 % had hands free of sores. The mean ± sd food safety and hygienic practice scores of the SFVs were 11⋅23 ± 2⋅67 with a minimum score of 2 points and a maximum score of 14 points (Fig. 1).

### Univariate analysis of factors associated with food safety and hygienic practice scores

The mean practice scores of the participants were stratified by general and demographic characteristics and presented in Table 4. There were differences in the mean (sd) practice scores for vendors who had a high level of education (*M* = 11⋅6, sd = 2⋅17) and those with no formal education or low level of education (*M* = 10⋅26, sd = 2⋅86). Male vendors had higher mean (sd) practice scores of 12⋅26(2⋅09) compared to their female counterparts who had (*M* = 11⋅41, sd = 2⋅79).

**Table 4.** Mean (sd) food safety and hygienic practice score stratified by general and socio-demographic characteristics

Variable	*N*	Mean (sd)	95 % CI		*P*-value
Education					
None	122	10⋅29(2⋅86)	9⋅77	10⋅8	0⋅001
Low Education	216	11⋅76(2⋅36)	11⋅44	12⋅07	
High Education	86	11⋅61(2⋅17)	12⋅64	13⋅57	
What happens to leftover food					
Given away	104	12⋅2(2⋅43)	11⋅84	12⋅79	0⋅018
Sold the next day	114	11⋅37(2⋅85)	10⋅84	11⋅9	
Taken home to eat	167	11⋅34(2⋅66)	10⋅93	11⋅74	
Throw away	39	11⋅59(2⋅44)	10⋅8	12⋅38	
Who is the owner					
Joint Owner	73	11⋅59(2⋅41)	11⋅03	12⋅15	0⋅744
Non owner	77	11⋅82(2⋅74)	11⋅2	12⋅44	
Owner	274	11⋅55(2⋅71)	11⋅23	11⋅88	
Who cooks the food					
Employee	65	12⋅05(2⋅53)	11⋅42	12⋅67	0⋅136
Mix	129	11⋅47(2⋅66)	11	11⋅93	
Self	219	11⋅49(2⋅73)	11⋅13	11⋅86	
Spouse	11	13(1⋅18)	12⋅21	13⋅79	
Gender					
Male	70	12⋅61(2⋅09)			0⋅001
Female	354	11⋅41(2⋅72)			

Mobile	75	12⋅77(2⋅26)			<0⋅01
Stationary	349	11⋅36(2⋅68)			
Medical check					
No		11⋅66(2⋅6)			0⋅489
Yes		11⋅43(2⋅88)			
Where is food cook					
Home	216	11⋅2(2⋅62)			0⋅002
Site	208	12⋅02(2⋅64)			
Marital status					
Single	174	11⋅89(2⋅64)			0⋅469
Married	250	11⋅42(2⋅67)			
Business certificate					
No	372	11⋅35(2⋅63)			<0⋅01
Yes	52	13⋅42(2⋅15)			
Nutrition training					
No	242	11⋅75(2⋅47)			0⋅214
Yes	182	11⋅42(2⋅89)			
Safety training					
No	224	11⋅24(2⋅67)			0⋅002
Yes	200	12⋅02(2⋅6)			

### Correlation analysis among continuous variables and food hygiene and safety practice scores

A weak negative correlation (*r* = −0⋅100) was observed between food safety practices and the number of days food vendors worked per week (Table 5). A negative correlation (*r* = −0⋅116) was observed between the food vendor's age and food safety practice.

**Table 5.** Correlation analysis among related factors and food safety and hygienic practice score of food vendors

Variable	Age	Dw/W	Hw/D	FSP	FSK	FSA	NK	WE
Age	1⋅000							
Dw/W	−0⋅045	1⋅000						
Hw/D	0⋅197**	0⋅135**	1⋅000					
FSP	−0⋅236**	−0⋅100*	0⋅064	1⋅000				
FSK	0⋅116*	0⋅192**	−0⋅083	0⋅372**	1⋅000			
FSA	−0⋅095	−0⋅242**	−0⋅172**	0⋅422**	0⋅660**	1⋅000		
NK	−0⋅102*	0⋅111*	−0⋅163**	0⋅171**	0⋅529**	0⋅329**	1⋅000	
WE	0⋅363**	0⋅033	0⋅206**	−0⋅060	−0⋅029	−0⋅116*	0⋅165**	1⋅000

Dw/W, days worked per week; Hw/D, hours of work per day; FSK, food safety knowledge; FSA, food safety attitude; FSP, food safety practice; NK, nutrition knowledge; WE, workers employed.

* Correlation is significant at the 0⋅05 level (two-tailed).

** Correlation is significant at the 0⋅01 level (two-tailed).

### Multiple linear regression analysis of factors associated with food safety and hygienic practice scores

A multiple linear regression was run to determine factors associated with food safety and hygienic practice scores and the results are presented in Table 6. The factors explained 31⋅4 % of the variability of the dependent variable. The slope for no education [*B* = −2⋅10, CI (−2⋅84, −1⋅36), *P* < 0⋅001] and high education indicated that there was a difference between vendors with low education and those with high education. Participants with low education scored 2⋅1 points lower on average as compared to those with higher education with respect to food safety and hygienic practice score.

**Table 6.** Multiple linear regression analysis of factors associated with food safety and hygienic practice scores

Variable	*B*	95 % CI	*β*	*T*	*P*-value
(Constant)	9⋅84	6⋅73–13⋅0		6⋅23	<0⋅001
Gender					
Male	0⋅55	−0⋅10 to 1⋅20	0⋅08	1⋅67	0⋅095
Age	−0⋅03	−0⋅06 to 0⋅00	−0⋅10	−1⋅88	0⋅061
Marital status					
Single	0⋅04	−0⋅46 to 0⋅54	0⋅01	0⋅15	0⋅883
No. of workers employed	0⋅07	−0⋅02 to 0⋅17	0⋅08	1⋅50	0⋅135
No. of days worked per week	−0⋅12	−0⋅35 to 0⋅10	−0⋅05	−1⋅06	0⋅291
No. of hours of work	0⋅13	0⋅05–0⋅20	0⋅15	3⋅14	0⋅002
Food hygiene and safety knowledge score	0⋅18	0⋅07–0⋅29	0⋅21	3⋅20	0⋅002
Nutrition knowledge score	−0⋅07	−0⋅22 to 0⋅08	−0⋅04	−0⋅89	0⋅377
Attitude score	0⋅04	0⋅01–0⋅07	0⋅14	2⋅74	0⋅006
Level of education					
None	−2⋅10	−2⋅84 to −1⋅36	−0⋅36	−5⋅57	<0⋅001
Low	−0⋅82	−1⋅45 to −0⋅20	−0⋅15	−2⋅59	0⋅010
Stationary/Mobile					
Stationary	0⋅91	0⋅31–1⋅50	0⋅13	2⋅98	0⋅003
Safety training					
Has not received food safety training	−0⋅05	−0⋅56 to 0⋅45	−0⋅01	−0⋅18	0⋅855
Nutrition training					
Has not received nutrition training	−0⋅10	−0⋅66 to 0⋅46	−0⋅02	−0⋅36	0⋅721
Business certificate					
Has no business certificate	−1⋅21	−2⋅03 to −0⋅39	−0⋅15	−2⋅89	0⋅004
Has undergone medical check					
No	0⋅68	0⋅07−1⋅29	0⋅11	2⋅19	0⋅029
Business owner					
Owner	0⋅05	−0⋅59 to 0⋅69	0⋅01	0⋅15	0⋅885
Joint owner	0⋅17	−0⋅64 to 0⋅97	0⋅02	0⋅41	0⋅884
Place of cooking food					
Home	−0⋅59	−1⋅04 to 0⋅14	−0⋅11	−2⋅58	0⋅010
Who cooks the food					
Self	0⋅38	−1⋅12 to 1⋅87	0⋅07	0⋅50	0⋅620
Mix	0⋅60	−0⋅94 to 2⋅15	0⋅11	0⋅77	0⋅442
Employee	−0⋅01	−1⋅52 to 1⋅50	−0⋅00	−0⋅01	0⋅992
What happens to leftover					
Given away	0⋅59	−0⋅33 to 1⋅51	0⋅10	1⋅27	0⋅205
Sold the next day	0⋅48	0⋅46 to 1⋅43	0⋅08	1⋅00	0⋅317
Taken home	0⋅29	−0⋅58 to 1⋅16	0⋅05	0⋅65	0⋅514

*F* (25, 398) = 8⋅75, *P* < 0⋅0005, *R*^2^ = 0⋅314.

## Discussion

The present study evaluated the nutrition knowledge, attitude and food safety and hygienic practices of SFVs in Northern Ghana. The nutrition-related knowledge was generally good although some notable deficiencies were identified. The food vendors’ attitude towards food safety and hygienic practices was commendable. However, several of the actual food handling techniques by street vendors posed severe concerns.

According to the present study, over 60 % of the participants were able to identify the various food nutrients, their functions and food sources. These findings are similar to the findings of Spronk *et al.*^(12)^ in Ethiopia which found 72⋅1 % of the participants knew that carbohydrate gives energy to the body whiles 75⋅9 % identified blood-giving foods and 55⋅6 % knew what was a balanced diet.

The present study assessed food vendors’ knowledge about ways by which food can be contaminated, where 87⋅3 % of the participants said poor personal hygiene could lead to food contamination. These findings were consistent with those of a study conducted in Bangladesh by Hossen *et al*.^(13)^ where 99 % of the participants agreed that poor personal hygiene may increase the risk of foodborne illness. These similarities could be a result of the similar educational backgrounds of the participants in the respective studies.

Another important finding of the present study was that we did not establish an association between the nutrition-related knowledge and food safety and hygienic practice of SFVs despite their relatively high overall nutrition-related knowledge. This could be ascribed to ineffective supervision by government agencies to ensure compliance with standard practices.

The present study showed that the attitude of SFVs towards food safety and hygienic practices was generally positive. This finding is similar to those of previous studies. A study conducted by Iwu *et al*.^(3)^ in Nigeria revealed that behaviour was significantly associated with the level of food hygienic practice. According to a study conducted by Auad *et al*.^(14)^ in Brazil, there is a significant impact of food safety training on attitudes. This is not surprising as one would expect that high scores on attitude would lead to good practice. One would observe that most of the participants in the present study had some level of education, which may explain the high score of participants since formal education has the possibility of improving attitude.

We found a weak negative correlation (*r* = −0⋅100) between food safety practice scores and the number of days food vendors worked per week. This could be due to the fact that the continuous engagement of SFVs over the years, makes one become too familiar with the job which may affect their efforts of doing the right thing since they may take things for granted as their practices are not regularly monitored. Despite the income generated as a result of continuous sales of food, food vendors need to prioritise the safety of the public and adopt safe hygienic practices.

One of the study's noteworthy findings was that SFVs who had attained a high level of education had higher practice scores compared to their counterparts who had a low level of education. This finding is similar to those reported by Hossen *et al*.^(13)^ among food vendors in Bangladesh, where the level of education had a substantial impact on the vendors’ food safety and hygiene procedures. Most food preparation skills, personal hygiene and cleanliness are learned from friends, instructors and the media, and these could be the reason for the present finding. However, a low level of education diminishes awareness, whereas a higher level of education improves understanding, which influences one's attitude and, eventually, hygiene behaviours. It means that SFVs should be encouraged to obtain at least a basic level of education before pursuing a career in catering services.

The present study showed that SFVs who had gone through a medical check-up before starting their business had an increased practice score compared to those who did not undertake medical check-up before commencing their business. Studies conducted by Adane *et al*.^(15)^ and Azanaw *et al*.^(16)^ both from Ethiopia supports this finding by revealing in their respective studies that medical check-up was significantly associated with good levels of food hygiene and safety practice by food handlers. This could be credited to the fact that the vendors are conscious of the safety of the food they sell in order to promote public health.

Furthermore, the study revealed that SFVs who had a business certificate had an increased practice score compared to their colleagues who had no business certificate. This can be attributed to the fact that they followed the protocols of the business given to them during the process of higher business registration. This was supported by a study conducted by Addo-Tham *et al*.^(10)^ in Ghana that revealed that food safety practices were associated with license status.

In the present study, there was a negative relationship between the number of working hours and food safety and hygienic practice scores. This may as a result of fatigue during long working hours which may lead to not observing some of the protocols of food safety and hygienic practices.

An important finding of the present study was that food safety knowledge scores were positively and significantly associated with the food safety and hygienic practice scores of the participants. This may be attributed to the fact that vendors with higher knowledge may have better education and training in food safety. Several studies have previously reported a positive association between the level of awareness, attitude and food safety training with SFVs’ hygienic practices^(17–19)^.

Again, SFVs’ attitudes showed a positive association with their food safety and hygienic practice scores. These findings were similar to a study conducted by Iwu *et al*.^(3)^ in Nigeria which found that food vendors’ attitudes were substantially linked to their level of hygienic standards. This may be due to their level of food safety knowledge, as well as the type of food safety training courses they have undergone.

This present study contributes new knowledge on the topic, particularly in Northern Ghana, where studies on the topic are scarce. The findings could be useful for providing targeted education to food vendors in Ghana and other parts of the world with similar settings.

The present study is limited by its cross-sectional nature as this design does not allow for the determination of causality. In addition, the use of self-reports makes the findings liable to social desirability and recall biases. Its generalisability is also affected by the fact that it was conducted in a single setting. Also, the questionnaire used for this study was not standardised; however, we believe it was able to measure our intended objectives as pre-testing was performed on ten SFVs and feedback was used to improve the overall quality of the questionnaire. Moreover, the scarcity of studies on nutrition knowledge was another limitation of the study. Notwithstanding, the findings of this study provide important themes and topics that could be useful for the design of training interventions to create awareness among SFVs regarding food safety and hygienic practice.

## Conclusion

The nutrition-related knowledge of the participants was generally adequate although some notable deficiencies were identified, and their food safety and hygienic practices were less desirable. Food safety and hygienic practice was influenced by education level, having a business certificate, number of hours worked, having medical check-up, mode of operation of food vending business (stationary or mobile) and food hygiene and safety knowledge. The regulatory authorities should increase their frequency of checking for compliance to ensure that SFVs put their knowledge and attitudes into actual practice to safeguard consumers. Future studies should be interested in investigating the nutrient quality and diversity of street foods and their potential in helping meet the nutritional needs of the population, especially the urban poor.
